# Reproductive Hazards Still Persist in the Microelectronics Industry: Increased Risk of Spontaneous Abortion and Menstrual Aberration among Female Workers in the Microelectronics Industry in South Korea

**DOI:** 10.1371/journal.pone.0123679

**Published:** 2015-05-04

**Authors:** Inah Kim, Myoung-Hee Kim, Sinye Lim

**Affiliations:** 1 Department of Occupational and Environmental Medicine, College of Medicine, Hanyang University, Seoul, Republic of Korea; 2 People’s Health Institute, Seoul, Republic of Korea; 3 Department of Occupational and Environmental Medicine, College of Medicine, Kyung Hee University, Seoul, Republic of Korea; University of Tennessee Health Science Center, UNITED STATES

## Abstract

**Objectives:**

Despite the global expansion of supply chains and changes to the production process, few studies since the mid-1990s and 2000s have examined reproductive risks of the microelectronics industry; we examined the reproductive risks among female microelectronics workers in South Korea.

**Methods:**

Based on claim data from the National Health Insurance (2008–2012), we estimated age-specific rates of spontaneous abortion (SAB) and menstrual aberration (MA) among women aged 20 to 39 years. We compared data between microelectronics workers and three different control groups: economically inactive women, the working population as a whole, and workers employed in the bank industry. For an effect measure, age-stratified relative risks (RRs) were estimated.

**Results:**

Female workers in the microelectronics industry showed significantly higher risk for SAB and MA compared to control groups. The RRs for SAB with reference to economically inactive women, working population, and bank workers in their twenties were 1.57, 1.40, and 1.37, respectively, and the RRs for MA among females in their twenties were 1.54, 1.38, and 1.48, respectively. For women in their thirties, RRs for SAB were 1.58, 1.67, and 1.13, and those for MA were 1.25, 1.35, and 1.23 compared to the three control populations, respectively. All RRs were statistically significant at a level of 0.05, except for the SAB case comparison with bank workers in their thirties.

**Conclusions:**

Despite technical innovations and health and safety measures, female workers in microelectronics industry in South Korea have high rates of SAB and MA, suggesting continued exposure to reproductive hazards. Further etiologic studies based on primary data collection and careful surveillance are required to confirm these results.

## Introduction

Reproductive health is among the most important occupational health issues for both men and women, mainly because the consequences extend beyond the workers themselves to their offspring, in the form of increased risk of congenital anomalies or cancer [1,2]. The health outcomes of reproductive toxicity are broadly categorized into two groups: (a) developmental outcomes such as clinical spontaneous abortion (hereafter, SAB), subclinical fetal loss, congenital anomalies, fetal growth retardation, or fetal/neonatal/infant death, and (b) reproductive outcomes including infertility, prolonged time to conception, or menstrual aberration (hereafter, MA) [3].

Many occupational hazards, including exposure to (a) chemicals such as pesticides, organic solvents, metals, anesthetic gases, or antineoplastic drugs, (b) physical conditions like ionizing radiation, or (c) ergonomic and psychosocial factors such as heavy workloads, long working hours, night shifts, or job stress are known to be associated with various adverse reproductive outcomes [4–6].

Regarding reproductive health, special attention should be paid to the microelectronics industry, which produces semiconductors or printed circuit boards, for two reasons. First, this industry uses a lot of potential repro-toxic agents. According to the historical hygiene assessment in a semiconductor company by the U.K. Health and Safety Executive, workers could be exposed to various repro-toxic agents, including lead, nickel, arsenic and arsenical compounds, tetrachloroethylene, trichloroethylene, or other aliphatic hydrocarbon solvents, ionizing radiation, or shift work [7]. Such concern was supported by empirical evidence. A series of studies published in 1990s and 2000s from the U.S. and Taiwan reported increased risks of adverse reproductive outcomes among the microelectronics workers, including SAB [8–13], infertility/subfertility [12,14,15], MA [16,17], and birth defects or cancer of offspring [1,2,18]. In case of South Korea (hereafter, Korea), public first recognized such a danger in 1995, when 16 of 25 female workers exposed to solvents containing 2-bromopropane in one electronics company developed secondary amenorrhea [19]. Second, globally, many of manufacturing workers in the microelectronics industry are women [20], mostly in their childbearing age. In Korea, the microelectronics industry is among the few manufacturing industries to hire a great number of female workers. With the growth of product markets and the expansion of global supply chain, more workers may be employed in this industry, especially in developing countries where wage costs are lower and labor rights are less protected [21].

Despite its critical consequence and a huge size of workers potentially affected, knowledge about current situation in the microelectronics is very limited; nearly all the previous studies evaluating reproductive risk in the microelectronics came from the U.S. and Taiwan and covered the period between 1970 and the 2000s. In Korea, few studies have been reported since the abovementioned incident [19], but none was conducted in the microelectronics industry [22–25]. Since then, working conditions have considerably changed. For example, ethylene glycol ether (hereafter EGE), a well-known chemical responsible for adverse reproductive outcomes has been phased out [26]. Also many parts of dangerous process have been automated; for instance, workers from a semiconductor company of Korea recalled that fabrication process was not sealed and they routinely handled chemicals and devices with their hands [27], which is not the case today. On the other hand, new materials and work processes have been continuously introduced by rapid technical development, of which health impacts are rarely known [20]. Currently, there is little information whether the reproductive risk has declined or not in this industry, although debates on cancer risk—in particular, hematologic malignancies—among semiconductor workers in Korea have been lively over the last decade [28–31].

In this context, our study aimed to fill the evidence gap in this field. Based on secondary data analysis, we examined the current reproductive risks with a focus on SAB and MA among female workers in the microelectronics industry in Korea.

## Methods

### Study subjects

From claim data of the National Health Insurance (hereafter, NHI) program, we obtained the annual numbers of women aged 20 to 39 years who had ever utilized healthcare services for SAB and MA, between 2008 and 2012. The NHI is a single-payer social insurance program, managed by the national government. It covers 97% of population, with the remaining 3% covered by the Medical Care Assistance program. The insured (= insurance holders) are classified into two groups; employees or employers, and the self-employed. Individuals who do not have their own income and are supported mainly by the former group are designated as ‘dependents’ [32]. Total population of women aged 20 to 39 were 7,270,163 in 2011, and 37.0% and 34.6% of them were holders and dependents of employment-based NHI program, respectively [33].

The ‘exposed’ group included all female workers employed in the three largest microelectronics corporations in Korea. The annual numbers of treated individuals and employees varied slightly across years. The average annual number of female workers over the five years was 38,822, while based on the 2010 census, a total of 122,563 females aged 20 to 39 were estimated to work in the microelectronics industry as a whole [34]. According to the Repository of Korea’s Corporate Filings of Financial Supervisory Service in 2013, 41,672 female workers were employed by these three companies, with 61.7% of them hired by the largest company and 23.3% by the second largest one [35].

For comparison, three different control groups were selected under different counter-factual assumptions. The first control group included all female dependents of the employment-based NHI program. They were in economically inactive states and supposed to be not exposed to any occupational hazards. The average number of women in this group over the 5 years was 2,413,830. The second control group included all female holders of employment-based NHI program, who were actively working across all industries. This group, which included an average annual number of 2,473,938, was selected to ensure that healthy worker effects were taken into account in the data analyses. Finally, the third control group included female workers employed in four major banks, who were supposed to be exposed to psychosocial stress, but not to chemical or physical hazards and night work. This group included 21,060 individuals on average over the 5-year period, while the 2010 census estimated that 108,846 women aged 20 to 39 worked in the banking and finance industry [34].

### Reproductive outcomes

Two reproductive outcomes, SAB and MA, were considered. According to the 6^th^ version of the Korean Standard Classification of Disease and Causes of Death, the code ‘O03’ represents SAB in various forms. MA included secondary amenorrhea (N91.1 and N91.2), secondary oligo- and hypo-menorrhea (N91.4 and N91.5), excessive and frequent menstruation with regular cycles (N92.0), excessive and frequent menstruation with irregular cycles (N92.1), other specified irregular menstruation (N92.5), and unspecified irregular menstruation (N92.6).

All the cases were identified from NHI claim data, and therefore unrecognized early fetal loss or cases without medical utilization could not be included in the analysis. In addition, misdiagnosis or coding errors may have been possible, although it is unlikely that such misclassification occurred in different proportions among groups.

Since there is no such a thing like ‘chronic’ SAB status, the number of SAB cases would correspond to annual incidence. It is possible for a woman to experience repeated SAB events within a year or through five years. The former case was considered as ‘one’ individual, while the latter was treated as a separate case. Meanwhile, MA is not an acute event of which beginning or ending could not be pinpointed. So, the number of MA cases would correspond to interval prevalence.

### Statistical analysis

The data were provided to the authors during parliamentary inspection, in the form of aggregated data without any individual information in order to protect patient privacy. The only information available was the numbers of treated cases and population denominators by year, age (2-year interval), NHI premium level (quartiles), affiliated industry, and holder status.

We further aggregated data over 5 years because the annual numbers of treated individuals remained small, in particular those of SAB cases in the microelectronics and banking industry. In order to control for an age effect, we stratified the sample into two groups based on age: 20–29 and 30–39 years. Socioeconomic status measured by the NHI premium quartiles was not considered in this analysis, as no difference across groups was observed in a preliminary analysis.

We calculated group-specific rates with 95% confidence intervals (CI) under the assumption of the Poisson distribution and estimated relative risks (hereafter, RRs) for the microelectronics industry with reference to the three different control groups.

## Results

The annual numbers of treated cases and denominators for each group are shown in Table 1. Overall, MA was more common than SAB. The annual rates of SAB ranged from 1.72 to 3.20 per 1,000 across all groups over the 5-year period, with higher rates observed in the exposed group than in the control groups. For MA, the annual rates varied from 44.35 to 84.59 per 1,000 across all groups over the 5-year period, with higher rates noted in the exposed group compared to the control groups (Table 1).

**Table 1:** Annual numbers and rates per 1,000 for spontaneous abortion and menstrual aberration according to patient group, 2008–2012.

Group		Year	Number of individuals	Spontaneous abortion (SA)		Menstrual aberration (MA)	
				Number of cases	Rate per 1,000 (95% CI)	Number of cases	Rate per 1,000 (95% CI)
Exposed group	Microelectronics workers	2008	39,388	82	2.08(1.68-2.59)	3,122	79.26(76.53-82.09)
		2009	37,816	88	2.33(1.89-2.87)	3,090	81.71(78.88-84.64)
		2010	39,838	87	2.18(1.77-2.7)	3,015	75.68(73.03-78.43)
		2011	40,902	131	3.2(2.7-3.8)	3,460	84.59(81.82-87.46)
		2012	36,168	110	3.04(2.52-3.67)	2,917	80.65(77.78-83.63)
Control groups	Dependents of employment-based NHI	2008	24,82,176	4,764	1.92(1.87-1.98)	1,21,808	49.07(48.8-49.35)
		2009	24,25,842	4,481	1.85(1.79-1.9)	1,19,399	49.22(48.94-49.5)
		2010	24,25,030	4,275	1.76(1.71-1.82)	1,15,403	47.59(47.31-47.86)
		2011	23,70,691	4,235	1.79(1.73-1.84)	1,17,619	49.61(49.33-49.9)
		2012	23,65,410	5,200	2.2(2.14-2.26)	1,23,248	52.1(51.81-52.4)
	Holders of employment-based NHI	2008	23,66,827	4,561	1.93(1.87-1.98)	1,20,925	51.09(50.8-51.38)
		2009	24,21,571	4,160	1.72(1.67-1.77)	1,19,685	49.42(49.15-49.71)
		2010	24,74,351	4,502	1.82(1.77-1.87)	1,25,032	50.53(50.25-50.81)
		2011	25,31,671	4,565	1.8(1.75-1.86)	1,32,556	52.36(52.08-52.64)
		2012	25,75,268	5,571	2.16(2.11-2.22)	1,44,595	56.15(55.86-56.44)
	Bank workers	2008	24,194	48	1.98(1.5-2.63)	1,073	44.35(41.77-47.08)
		2009	22,155	49	2.21(1.67-2.93)	1,116	50.37(47.5-53.42)
		2010	20,405	40	1.96(1.44-2.67)	1,095	53.66(50.58-56.94)
		2011	19,602	57	2.91(2.24-3.77)	990	50.51(47.46-53.75)
		2012	18,945	55	2.9(2.23-3.78)	1,070	56.48(53.19-59.97)

Based on the 5-year aggregated data, we found that SAB was more frequent among women in their 30s, while MA was more common among women in their 20s across all comparison groups. The microelectronics workers in their 20s and 30s were more likely to have medical utilization for SAB and MA, compared to the three control groups in the same age. Among women in their 20s, dependents of employment-based NHI (economically inactive population) were least likely to experience SAB and MA. Meanwhile, holders of employment-based NHI (wage earners as a whole) had the lowest rates for SAB and MA among women in their 30s (Table 2).

**Table 2:** Age-specific rates (per 1,000) of spontaneous abortion and menstrual aberration by group, based on aggregated data from 2008–2012.

Age	Group		Number of individuals	Spontaneous abortion (SA)		Menstrual aberration (MA)	
				Number of cases	Rate per 1,000 (95% CI)	Number of cases	Rate per 1,000 (95% CI)
20~29	Exposed	Microelectronics workers	1,48,247	331	2.23(2.01-2.49)	13,130	88.57(87.07-90.1)
	Control groups	Dependents of employment-based NHI	53,52,347	7,485	1.4(1.37-1.43)	3,07,534	57.46(57.26-57.66)
		Holders of employment-based NHI	61,73,723	9,861	1.6(1.57-1.63)	3,95,786	64.11(63.91-64.31)
		Bank workers	45,199	89	1.97(1.6-2.42)	2,697	59.67(57.46-61.96)
30~39	Exposed	Microelectronics workers	45,865	167	3.64(3.13-4.24)	2,474	53.94(51.86-56.11)
	Control groups	Dependents of employment-based NHI	67,16,802	15,470	2.3(2.27-2.34)	2,89,943	43.17(43.01-43.32)
		Holders of employment-based NHI	61,95,965	13,498	2.18(2.14-2.22)	2,47,007	39.87(39.71-40.02)
		Bank workers	60,102	160	2.66(2.28-3.11)	2,647	44.04(42.4-45.75)

The RRs for SAB and MA of the microelectronics workers with reference to the three different control groups are shown in Table 3. The risks for SAB of the microelectronics workers in their 20s were 1.57 (95% CI 1.43–1.78), 1.40 (95% CI 1.25–1.56), and 1.37 (95% CI 1.10–1.70) times higher compared to dependents of employment-based NHI, holders of employment-based NHI, and bank workers, respectively. Such risk elevation was also clear among those aged 30–39 years: the RRs with reference to dependents of employment-based NHI and holders of employment-based NHI were 1.58 (95% CI 1.36–1.84) and 1.67 (95% CI 1.44–1.95), respectively. The relative difference with bank workers was not statistically significant (RR = 1.13, 95% CI 0.90–1.43).

For MA, compared to dependents of employment-based NHI, holders of employment-based NHI, and bank workers, the RRs of microelectronics workers in their 20s were 1.54 (95% CI 1.52–1.57), 1.38 (95% CI 1.36–1.41), and 1.48 (95% CI 1.43–1.55), respectively. Risk elevation compared to three different controls was also observed among those aged 30–30 years (Table 3).

**Table 3:** Relative risks for spontaneous abortion and menstrual aberration of microelectronics workers compared to three different control groups, based on aggregated data from 2008–2012.

Outcome	Age	Comparison	Relative risk (95% CI)
Spontaneous abortion (SA)	20~29	vs. dependent of employment-based NHI	1.57(1.43-1.78)
		vs. holders of employment-based NHI	1.4(1.25-1.56)
		vs. bank workers	1.37(1.1-1.7)
	30~39	vs. dependents of employment-based NHI	1.58(1.36-1.84)
		vs. holders of employment-based NHI	1.67(1.44-1.95)
		vs. bank workers	1.13(0.9-1.43)
Menstrual aberration (MA)	20~29	vs. dependent of employment-based NHI	1.54(1.52-1.57)
		vs. holders of employment-based NHI	1.38(1.4-1.41)
		vs. bank workers	1.48(1.43-1.55)
	30~39	vs. dependent of employment-based NHI	1.25(1.2-1.3)
		vs. holders of employment-based NHI	1.35(1.3-1.41)
		vs. bank workers	1.23(1.16-1.29)

## Discussion

Using the NHI claim data over the period between 2008 and 2012, we found that female workers employed in the microelectronics industry were more likely to undergo medical treatment for SAB and MA compared to three different control groups; (a) economically inactive women (dependents of employment-based NHI), (b) wage earners as a whole (holders of employment-based NHI), and (c) clerical workers employed in banks. Female workers in the microelectronics industry received approximately 13~67% more medical attention due to SAB compared to age-matched control groups. In addition, healthcare utilization for MA was more frequent among the microelectronics workers by 23~54% compared to control groups.

Our analysis was based on the NHI claim data, which means that cases were identified only if a patient used healthcare services. Untreated cases such as unrecognized early fetal loss or MA with mild symptoms were not included. It is plausible to assume that wage earners would seek medical attention less for mild symptoms or be actually healthy (i.e., healthy worker effects). It should also be noted that the population at risk for SAB could not be clearly defined in our data. It is desirable that sexually active women who do not use contraceptive measures or those who have clinically recognized pregnancies are defined as ‘at-risk’ population for SAB. Such information was not available from the NHI data. Nevertheless, it is reasonable to assume that the relative size of such at-risk population would be greater in economically inactive women than in wage earners, which implies that the SAB rates may be relatively underestimated among the working population. In fact, one Korean study reported that the SAB rate among clinically recognized pregnancies was 4.54% and 6.65% among female dependents and female holders of employment-based NHI, respectively [36]. In addition, there could be misclassification in case ascertainment, since these codes are assigned by healthcare providers for their reimbursement not for research. However, there is no reason to believe that such misclassification occurred to different extents among comparison groups.

Another important limitation is that information on specific exposure or past occupational history was not available. In the microelectronics industry (“exposed” group), we could not identify particular workers who were potentially exposed to reproductive hazards or working in a specific position. There are many different jobs and work processes in the microelectronics industry, including the fabrication process, circuit board production, assembly of components, packaging, and office work. As such, workers are exposed to different risk factors depending on their position. By aggregating all workers in different positions into one “exposed” group, the potential risk related to working conditions of the microelectronics is likely to have been underestimated.

All of these limitations seem to bias the relative risk estimates toward the null, which contributes to conservative conclusions.

Although we could not estimate the absolute risk nor identify specific factors responsible for adverse reproductive outcomes because of the abovementioned limitations, our results indicate an overall increase of relative risk among workers in the microelectronics industry compared to the control groups.

Our results can be explained as follows. First, the elevated risk of the microelectronics workers relative to the non-working population could be considered as a combined effect of occupational hazards and working life *per se*. Meanwhile, these estimates might have been biased by healthy worker effect or infertility worker effect [37]. The former is associated with positive health selection among workers, resulting in underestimation of work-related hazardous effects. Infertile worker effect is a phenomenon that infertile or childless female workers choose to stay on their jobs, resulting in overestimation of work-related reproductive risk. However, our comparison within working population could minimize healthy worker effects as well as infertility worker effect. Still, the RR might be underestimated due to healthy worker effects in this comparison. The second comparison with working population as a whole could avoid such healthy worker effects. Still, the RR for SAB and MA in the microelectronics workers was significantly elevated, which means that specific exposures in the microelectronics might surpass the overall effects of various working conditions across all industries. Third, the microelectronics workers showed elevated risks compared to bank workers who may be exposed to psychosocial hazards such as job stress but not exposed to physical and chemical hazards or shift work. Finally, we should consider the possibility that elevated risk of SBA and MA among microelectronics workers might be confounded by health behaviors such as smoking or alcohol use [38]. However, such information was not available from the NHI claim data. According to the Korea National Health and Nutrition Examination Survey (2010–2012), smoking and drinking was more prevalent among working population compared to economically inactive women. In addition, smoking was more common in manual workers than in office workers, while drinking was so in office workers [39]. However, there are many different kinds of ‘manual’ jobs, and presumption of higher smoking prevalence among microelectronics workers is unreasonable. Rather, it is more reasonable to assume that microelectronics workers are less likely to be smokers because of their working conditions; workers have to wear cleanroom coveralls and take the air-shower entering the facility, because indoor dusts or particulates threaten the product quality. For its complicatedness and time pressure, even toilet use is considered as a burdensome project to workers [40]. In this context, it is unlikely that smoking and alcohol contributed to higher risk of SAB and MA among microelectronics workers, although there is no empirical evidence. From these findings, we could infer that hazardous working conditions specific to the microelectronics may contribute to the elevated risk for SAB and MA.

Meanwhile it should be noted that women in their 30s showed higher rates of SAB compared to their younger counterparts across all exposure groups. There could be three alternative explanations; infertile worker effect, exposure accumulation, and aging effect. Infertile worker effect seems to be valid for working population but is not relevant for economically inactive population. If exposure accumulation in microelectronics resulted in more SABs, then the relative risk estimated from comparison with various control groups would have been greater in older groups. However, the effect size of estimates was not so different between two age groups. In microelectronics, current exposure of chemicals during the first trimester of pregnancy might be more relevant for adverse reproductive outcomes, although heavy metals such as lead can be accumulated in body tissues and have repro-toxic effects [38]. Up to now, natural increase of SAB risk by aging might be the most reasonable explanation.

Although information on occupational exposure was not available, we could assume several factors responsible for the excess risk. Previous studies paid special attention to EGE regarding SAB and subfertility in the U.S. and Taiwan [9,12–15], while it is not certain whether EGE is still used in the microelectronics of Korea. However, recent hygiene assessments in the semiconductor industry in Korea identified several repro-toxic agents including arsenics, ionizing radiation, trichloroethylene, ethylene glycol, propylene glycol monomethyl ether, benzene, toluene, and xylene [29,41–43]. Other factors may play a role for adverse reproductive outcomes. According to a recent meta-analysis, long working hours of more than 40~52 hours per week, lifting more than 100 kg per day, or standing for more than 6 hours per day are significantly associated with miscarriage [44]. Manufacturing workers in the microelectronics industry of Korea usually work in 3-shift or 2-shift schedules, more than 40 hours per week, and in a standing position for a great part of working hours.

To the best of our knowledge, this is the first report showing an increased risk of SAB and MA among the microelectronics workers in Korea and also the only report based on the data of late 2000s in the microelectronics industry worldwide. Based on our findings, although information on specific hazards was not available, we could conclude that female workers in the microelectronics are still at an increased risk of SAB and MA, which may have been underestimated in this study due to biases toward the null. Despite technical innovations and health and safety measures by the microelectronics industry, workers appear to remain exposed to various reproductive hazards, at least in Korea. Given that our data came from the three biggest companies in Korea, it is plausible to assume that workers in small-sized companies of Korea or working in developing countries are more exposed to such risk. Adverse reproductive outcomes are serious in itself and also sentinels for other occupational health risk with a long latency or rare incidence, for example cancer [3,31]. Careful surveillance and further etiologic studies based on primary data collection and hygiene assessment are strongly recommended.
